# Shared work? Unravelling interspecies entanglements, agency, and the rhythms of equids at work

**DOI:** 10.3389/fvets.2025.1570879

**Published:** 2025-07-22

**Authors:** Tamlin Watson, Cara Clancy

**Affiliations:** The Donkey Sanctuary, Sidmouth, Devon, United Kingdom

**Keywords:** donkeys, working equids, working animals, work, labour, equid welfare, agency, more-than-human

## Abstract

Focusing on donkeys, this paper examines the type and scope of ‘work’ undertaken by working equids in three very different contexts in the United Kingdom, Europe and the Global South (case studies). Drawing on the concepts of ‘animal work’ and ‘nonhuman labour’ as discussed by critical theorists such as Porcher, Estebanez, Coulter, Barua and others we aim to: (i) Elaborate on the concept of ‘shared work’ by bringing key animal welfare concepts into dialogue with emerging literature on animal labour through a relational theoretical lens; (ii) Explore the nature of equid work including its physicality, and also the freedoms and opportunities that are afforded to equids (in terms of rest, play and kinship); (iii) Illustrate how work may be experienced by the equids themselves, using vignettes based on ‘more-than-human’ ethnographic fieldwork so as to foreground the equid perspective and illuminate questions of agency, sentience and subjectivity.

## Introduction

In recent years, animal theorists have begun to explore the concepts of ‘work’ and ‘labour’ in relation to working equids and their owners (1, 2). Other than a recent study by Geiger et al. (3) donkeys have largely been absent from the animal labour literature. Building on this, we focus on donkeys in this paper because (a) donkeys have a long history of working with and for humans throughout the world and (b) while there is significant literature on the welfare of working donkeys globally (4–7), this has yet to be brought into conversation with emerging literature on animal labour. Animal welfare discussions have historically been limited to somewhat reductive frameworks such as the Five Freedoms^1^ (8). But in more recent years, there has been a growing interest in more holistic/360′ approaches, including the Five Domains^2^ (9), the concepts of ‘One Health’^3^ (10) and ‘One Welfare’^4^ (11), all of which underline the interconnectedness of human/animal welfare, wellbeing and the environment. Appreciating complex interconnecting systems at play in a given context gives space to understand how situations, such as those for working equids, are not purely about individual animals but are a complex web of factors such as equid health, owner knowledge, the environment, and societal influences, all of which all conspire to influence wellbeing (12). The 3Fs^5^ provide a concise, simple summary of the three main overarching, essential equine needs, ‘friends, forage and freedom’, for ease of understanding and evaluation (13, 14). Increasing scholarly emphasis on ideas of ‘animal flourishing’ and ‘animal choice’ (15), along with the ever-expanding awareness of animal sentience (16) presents a rich opportunity to revisit the concepts and practices of animal work and nonhuman labour.

The notions of animal ‘work’ and ‘labour’ have been theorised in a range of contexts, including (eco)tourism and leisure (2, 17, 18), sport (19, 20), nature conservation and restoration (21–24), and across various industrial practices (1, 21, 25–28). While the terms ‘work’ and ‘labour’ are sometimes used interchangeably in the literature, nonhuman labour scholarship is generally more explicit in its positioning and emphasis on the commodification and capitalisation of nonhuman others [e.g., (17); see also (24)]. For ease and clarity and the desire to illuminate questions of animal welfare, we understand ‘animal work’ (29)^6^ as the practical activity or task the animal is engaged in, while ‘nonhuman labour’ here refers to the physical, mental and emotional effort or exertion on the part of the animal. Importantly, these are relational terms.

Work, and the labour that makes it possible, comprises a set of human-nonhuman relations and processes within specific socio-political and economic contexts. Those engaged in work are not fixed entities defined solely by the work activities they perform. Some critical animal theorists caution against the use of the category of ‘worker’ or ‘working animal’, i.e., the identification of an animal purely in relation to his/her work. For instance, Coulter (1) prefers to think of such animals as ‘animals who work’ and consider ‘work done by animals.’ For the sake of clarity and ease of language flow, we chose to use the terms ‘working equid’ and ‘working donkey’ in this paper but acknowledge that an animal is more than what he/she does for work. Entrenched hierarchies that demarcate and govern animal life are products of ideological and cultural constructs, including patriarchy, colonialism and essentialism (30–34). Both humans and animals suffer within systems of coercion and domination, as we go on to discuss. We therefore situate our work within feminist–posthumanist efforts to dispel hierarchies of life, relational distancing, alienation and estrangement between humans and animals. Haraway’s (35) concept of ‘becoming’ challenges binary distinctions by emphasising the interconnected relational nature of existence, the fluidity of identities and the transformational nature of interactions. The notion of ‘becoming’ (35) has been deployed in a limited number of studies on animal work (36) and we suggest it is helpful in this context insofar as it challenges ideas of humans and animals as fixed entities. As Geiger and Hovorka explore in their paper on working donkeys on in Botswana through ideas of ‘donkeying’ or ‘becoming donkey’ (37): “donkey subjectivity and donkey subject are embedded in spatiality or place-based relations of power. [they are] necessarily negotiated in and through the spatial dimensions of daily existence.” We are interested in ideas of animal welfare, sentience and subjectivity and this means looking beyond particular identities as working animals. What other modes of relation are taking place in these working contexts? How do practices of rest, play and kinship materialise (if at all) and what can they tell us about questions of nonhuman autonomy, agency and subjectivity?

To gain some insight into these questions, and the complex interactions with context, we will examine three different categories of work:

- Hard work—this is relentless, physically and physiologically demanding work where both working equid and handler may be constrained by socio-economic and societal factors, severely limiting agency.
- Decent work—the term ‘decent work’ was originally developed as a response to concerns about workers’ rights and labour standards (38). In this paper we use the term to denote work where equids and owners may have more freedom to choose the limits and pressures of their working day.
- Affective work—aimed to create or modify human emotional experiences such as the case with animal assisted therapy. In this paper equine assisted activities will be discussed, using a specific case study of donkeys. Equids working in this industry are expected to sustain heavy emotional labour. Equid agency in this context may vary depending on the ethos and approach of practitioners.

While most working equids are in the Global South (7, 39), donkeys still perform an important role within labour contexts in other countries further north (40, 41). Welfare conditions vary and, as we go on to discuss in this paper, this is largely dependent upon several factors including (i) economic circumstances, (ii) human welfare and wellbeing (iii) cultural or societal influences. Using examples drawn from our case studies, this paper acknowledges that some forms of work, when combined with a suitable environment and working conditions, can create positive experiences for humans and animals. Some work can be stimulating, even enjoyable, for both the animal and their human worker, owner or companion. For instance, Cobb et al. (42) assert that provision of meaningful (to the animal) opportunities to ‘exercise agency and increase behavioural diversity’ are essential for fulfilment and positive outcomes for animals. In the case of dogs, olfaction-based foraging activities were found to increase optimism and improve a dog’s welfare (43).

While instances of human/nonhuman companionship and cooperation do indeed exist in the context of work, this paper follows others who caution against the romanticisation of interspecies relations (18) and recognises the significant imbalance of power between animal workers and their human counterparts (44, 45). Working donkeys, particularly in poor and marginalised communities, often have extremely poor welfare and minimal agency (4, 46–48). Even in contexts where higher welfare exists, power dynamics still impact equids as they work, as we highlight in the case study on equine assisted activities. Taking a cautious approach with respect to ideas (and ideals) of mutuality and equality in the context of equid work, this paper develops and deploys the notion of ‘shared work’, which emphasises the relational aspects of human-animal work, drawing on relational geographies that highlight the many ways that nonhumans are connected to (and implicated in) human activity through matters of space and place (31, 49–52) and earlier theories that emphasise the webs of relations that exist between humans, plants, animals, microbes (30, 53–58).

In recent years the concept of ‘entanglement’ has expanded in the field of human-animal studies (59–63). As DeSilvey and Bartolini (64) suggest, ‘it has become commonplace to refer to the inextricable entanglement of human and nonhuman worlds’, for the world is comprised of ‘multitudes of lively agents [who] bring one another into being through entangled relations’. Humans and equids are entangled in shared work dynamics—and yet, the concept of entanglement has been underutilised in discussions on animal labour, and even less so in discussions around animal welfare and wellbeing, although the notions of ‘One Health’ (10) and ‘One Welfare’ (11) do arguably have philosophical leanings towards the notion of entanglement (65, 66). Entanglement and shared work capture the relational and spatial elements that are so important to subjective experiences, but too much emphasis on these can overlook animal agency within these contexts. Therefore, this paper is the first to bring together these various literatures to discuss the ways in which donkeys labour and how their entangled relationships with humans, the environment, and shifting economic landscapes have a bearing on how they experience this work and indeed how they might resist it—opening up reflections on animal autonomy and subjectivity.

Donkeys were domesticated over 7,000 years ago (67, 68) and have been used as working animals ever since. Being strong and surefooted, exhibiting great stamina in challenging arid environments, and requiring relatively low-calorie forage inputs (67, 68) made the donkey a perfect candidate for domestication (67, 69). Their natural ethology, or the study of their behaviour in natural conditions, reveals a high capacity for learning and problem-solving, while they also possess a well-developed sense of self-preservation (70). Donkeys are cautious by nature (71), which means they may resist tasks if they are forced to work in unfamiliar or in situations perceived as dangerous (72, 73). In addition, donkeys often mask signs of stress, pain, or fatigue which can lead handlers to inadvertently overwork them or fail to meet their needs leading to poor welfare (74, 75). Such practices not only harm these animals, leading to stress and fear responses when being handled but can also perpetuate a cycle of miscommunication and conflict (76). Incorporating ethological knowledge, including an appreciation that animals have cognitive and affective lives (77), enables some recognition of the animal experience (78). Appreciating that animals have consciousness, emotions and have agency does ‘provide a powerful case’ that animals are ‘worthy of moral concern’ (77), and highlights that consideration should be given to understand what they may need to live and thrive in their ‘own worlds’ (79). Haraway (35) eloquently states ‘caring means becoming subject to the unsettling obligation of curiosity, which requires knowing more at the end of the day than at the beginning’. Interactions between donkeys and people are multidirectional, both actors can respond to each other, and through these ‘intra-actions’ relationships get negotiated and renegotiated.

Today there are thought to be approximately 54 million donkeys globally (39), an unknown proportion of these will be working. Through their work, donkeys are essential to the survival of millions of people around the world, some of the most marginalised parts of society (6, 74, 80–84). This is why donkeys are highly pertinent to academic discussions on animal work and nonhuman labour. Despite being critical to the development of early pastoral societies, cities and, later, entire empires (67, 69, 85)—facilitating critical trade networks, contributing to agricultural development, construction and mining—donkeys have been largely overlooked in existing academic discussions on animal labour As such there are few articles specifically dedicated to donkeys as working animals, even fewer tackling the specific intricacies of their lives and, ultimately, their welfare. This paper is different insofar as it brings the animal labour literature into direct conversation with concepts of animal welfare and wellbeing, particularly the 3F’s, with a focus on working donkeys as marginalised species. Bringing together ideas of ‘animal work’ and ‘nonhuman labour’ into conversation with key concepts in animal welfare, including ‘freedom’ and ‘friendship’, this paper enables a novel articulation of ‘shared work’ such as what is ‘shared’ when equids work and, through this, we offer new insights into human-equid relations in the context of work, where the autonomy afforded to nonhuman actors may be marginal or limited and intimately entangled with social, political, economic and environmental factors.

## Methods

Given the limited discussions around how nonhuman labour is experienced by equids (including differences between human-equid working relationships and the factors influencing how work is managed), we identified the need for three ‘critical cases’ (86), chosen for the way they contribute to the exploration to the concept of ‘shared work’ and reflect the different qualities and characteristics of different types of equid work. The case studies highlight the complexities of what working life means for equids in three very different contexts:

1. ‘Hard work’—brick kilns in India.
2. ‘Decent work’—sensitive land management in Europe.
3. ‘Affective work’—equine assisted activities in the United Kingdom.

The case studies also shed light on the complex socio-economic and cultural factors influencing working equid welfare in these differing contexts. In addition, we use the case studies to delve into how these pressures and constraints influence the management, relationships, and agency of working equids in these situations. Finally, through the case studies, we explore how ‘work’ may be perceived by equids themselves, attending to their ethology and behaviour to guide understanding. Taking a ‘more-than-human’ lens (52), the authors work to emphasise equid voices and experiences while acknowledging the limits of human knowledge. Each case study is introduced through a piece of ethnographic writing in the form of a vignette based on real-life observations made by the researchers (87). The empirical material presented in this paper was gathered (by the authors) on separate occasions between 2018 and 2025, during in-depth fieldwork across multiple study sites. The material included observations of working equids and owners interacting, their reactions, interspecies communications and first-hand accounts from owners, and our own personal thoughts and reflections. All were recorded within detailed fieldnotes either chronicled in real-time on the sites during fieldwork or, if safety, time or other constraints necessitated, details were written away from field sites later that same day. The studies were conducted in accordance with the Declaration of Helsinki (88) and were approved by the Ethics Committee of The Donkey Sanctuary, project code number: TDS_I_2025-09-01. This consisted of surveys, interviews and observational data, and we revisited this existing data through the animal labour lens. Given that knowledge is always partial and situated (89) (particularly when it comes to nonhuman others), the authors have presented findings contextually, in relation to social, political, economic and environmental contexts. The case studies demonstrate that while there will always be methodological limitations, it is possible to offer real glimpses into the subjective, embodied, emotional, and relational experiences of nonhuman animals as they interact with humans. Through the case studies, the paper demonstrates why contextualised and situated accounts of equids at work proves useful when considering their welfare and wellbeing and provides a non-prescriptive framework or guide for thinking through the different characteristics and qualities of different types of equid work.

## Case studies

### Case study 1: ‘hard Work’ equid use in brick kilns in India

India, fieldnotes, 30 April 2018, AM.

**Figure 1:**
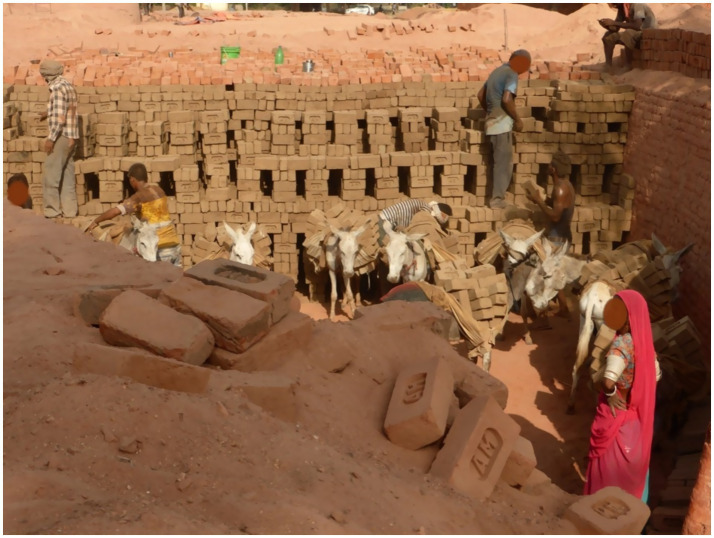
Donkeys waiting to be unloaded as the unfired bricks are stacked in an Indian brick kiln. Photo credit: TW.

*Its early in the morning and the heat is already stifling. The land inside the brick kiln is barren, any fertile topsoil has been removed exposing the clay subsoil beneath which is needed to mould the bricks. An occasional tree hangs on in isolation in an environment left barren and degraded from this industrial process. I can hear sounds of work; occasional shouts and the muffled hoof-fall of multiple animals. A high pile of bricks stretches for some distance, people stand here removing dusty, gritty bricks with their bare hands, placing them in careful order within the panniers strapped to each side of their donkeys’ backs. These are the green or unfired bricks, and once the panniers are full each donkey steadily picks themselves forwards in a well-trodden brick strewn route towards the kiln’s firing area. Bare-footed men, women and children take turns to drive the donkeys in a close procession to the kiln, sometimes resorting to using a stick to slap the donkeys’ hind quarters if deemed to be walking too slowly, although often this seems to be simply habitual. The kiln is a high brick lined area which is filled to the top with bricks in specific patterns allowing for ventilation (see* *Figure* *1**); once the area is filled the bricks will be covered with sand and a fire lit which will be drawn evenly through the vents.*

*Human and donkey workers have been performing this labour throughout the cooler hours of the night, rushing to finish the final quota before the day becomes unbearably hot. Eventually the quota is reached, and people and donkeys wearily make their way to the temporary brick-made shelters they call home whilst labouring in this kiln. The donkeys look exhausted, broken, their shuffling gaits an indication of the sheer hard toil they have endured. Each owner ties their donkeys along thick nylon lines on the ground outside their dwellings, there is no shade. Depending on the owner, and the personality of the donkeys, some donkeys may be able to reach each other for social contact but often they cannot. Donkeys considered too aggressive will not be allowed contact when tied up to rest, these donkeys are also hobbled when released for grazing so deprived of any further opportunity for social contact. One gentle, quiet, heavily scarred donkey stood to the side of an owner’s group of donkeys; he was clearly not being worked. When speaking to his owner, she told us ‘We cannot bear to leave him alone in our village when we leave for work, we worry he will be attacked by dogs or people. He has been with us for many years, he is part of our family, he will stay with us, we will not sell him’.*

*There is soft murmuring between families as they prepare a small fire to cook a one-pot breakfast and boil a kettle for their cups of chai. One family member walks along the tethered donkeys offering a small container of water to each, no donkey given the opportunity to drink to their fill. Once the donkeys are settled in their lines, the family gregariously eat their breakfasts, drink their chai and settle down on metal framed makeshift beds, their mattresses consisting of simple criss-crossed ropes woven across the frame. Babies are cocooned into small hammocks supported on simple triangular frames next to the adults’ beds, attached via a short section of fabric which is gently pulled to rock the babies to sleep.*

*As the afternoon sun loses some of its heat, the donkeys are offered a guided excursion to graze in neighbouring stubble fields, fields owned by neighbouring landowners. Donkeys shortly return to the worker’s dwelling areas, are given energy rich beans, grains, and sometimes ‘jaggery’ (sugar cane) for extra energy, they will need this meal to support them in the heavy work ahead. They are given water once more, as the owners eat their evening meals. As the day turns towards evening, the people, and their donkeys slowly walk across the baked, bare earth to begin their work shifts again.*

*The heat was already almost unbearable, and I became tired, hot and sweaty very quickly during interviews and observations. Spending time in brick kilns observing the heavy labour shared between these donkeys and their owners, one cannot help reflecting about how commodified both actors are and how desperate one would need to be to work in this industry. On leaving the kiln, I contemplated how it must feel for people and their donkeys to have no autonomy about how their daily lives are planned, and how it must feel for highly social species to have such limited opportunities for social and environmental interactions.*

The above excerpt was taken from field notes taken during a study in the brick kilns in Ahmedabad, Northern India. Donkeys are used in brick kilns to carry bricks requiring firing to and from kilns on site. Fired bricks are then sold off throughout India for construction projects. Equids are often used because most kiln layouts have narrow passages which only equids can easily pass through. The field notes were written by the researcher who was observing donkeys performing their final brick delivery rounds of the day before rest. Owners were interviewed shortly after donkeys were tied up and the owners were preparing to sleep for the day. Though some owners have kept donkeys for many years, others were new to this donkey business and may have only had their donkeys for one season (6). A community or peer-peer knowledge is relied upon by owners within these communities. However, the temporary nature of existence in the kilns means knowledge may not always be reliable or recognisable to people from other regions, so there is often a determined self-reliance, perhaps to avoid admission of a lack of knowledge to those unfamiliar, this can have consequences for their equids’ welfare (84, 90). A rarely noted additional impact of the brick kiln industry is the long-term degradation to the environment where kilns are located. In stripping off topsoil, polluting the land and compacting the subsoil to access the clay to make bricks, the industry leaves agricultural land impoverished, lacking in structure and fertility. This situation renders land unsuitable for agriculture, a situation that leaves the subsistence farmers, who often lease their land temporarily to brick kiln owners, unable to produce food (91, 92). These environments being unable to naturally regenerate are susceptible to further erosion, surface water run-off and landslides, particularly during monsoon seasons which are becoming ever more prevalent and prolonged with changes to climate (93).

Today, donkeys work in some of the lowest paid industries (mines, brick kilns, rubbish dumps, waste disposal industries)—doing what Coulter (1) describes as ‘dirty work’, which refers to work that is deemed degrading and/or undesirable. People also labour in these conditions and environments (94–98). Brick kiln workers are often in debt to the kiln owner of the kiln where they are bonded, debts are inherited by family members (47, 98). The brick kiln season lasts for 6 months, often people and their donkeys must travel long distances to reach the kiln where they may have been recruited to work by a labour scout or ‘jamadar’ employed by the kiln owner. Brick kiln workers are often entire families, forced to migrate due to their poverty and societal marginalisation (47, 84, 98, 99). Donkeys endure hard labour alongside people in the kilns, and they will only be released from this work if they become too old or infirm to continue, or if the owner finds other more lucrative work (6). As Narayanan (27) noted in her recent paper, ‘animal suffering shares characteristics with human suffering’ in these contexts, though animals have unique experiences as commodity labour, ‘which are inherent in being *not human*’.

Donkeys are most frequently used for labour in poor communities, and as such these equids have become connected with marginalisation, their status minimised within governmental institutions, ignored for health provision, ‘undervalued by wider society’ (3) and negatively impacted through derogatory societal discourse (100, 101). Whether working equids are given choice, have agency or even consent to participate in working activities is a moot point. Both human and equid actors often cohabit similarly challenging societal environments (80, 84) where they suffer their own forms of ‘social stratification’ (102), a process of ‘dual exploitation whereby all working class bodies—human and donkey—are subject to stark inequalities’ (37) within a socioeconomic, exploitative and coercive framework which limits and prejudices both (103).

### Case study 2: ‘decent work’ equid use in sensitive land management in Europe

Portugal, notes, 17 January 2025, AM.

**Figure 2:**
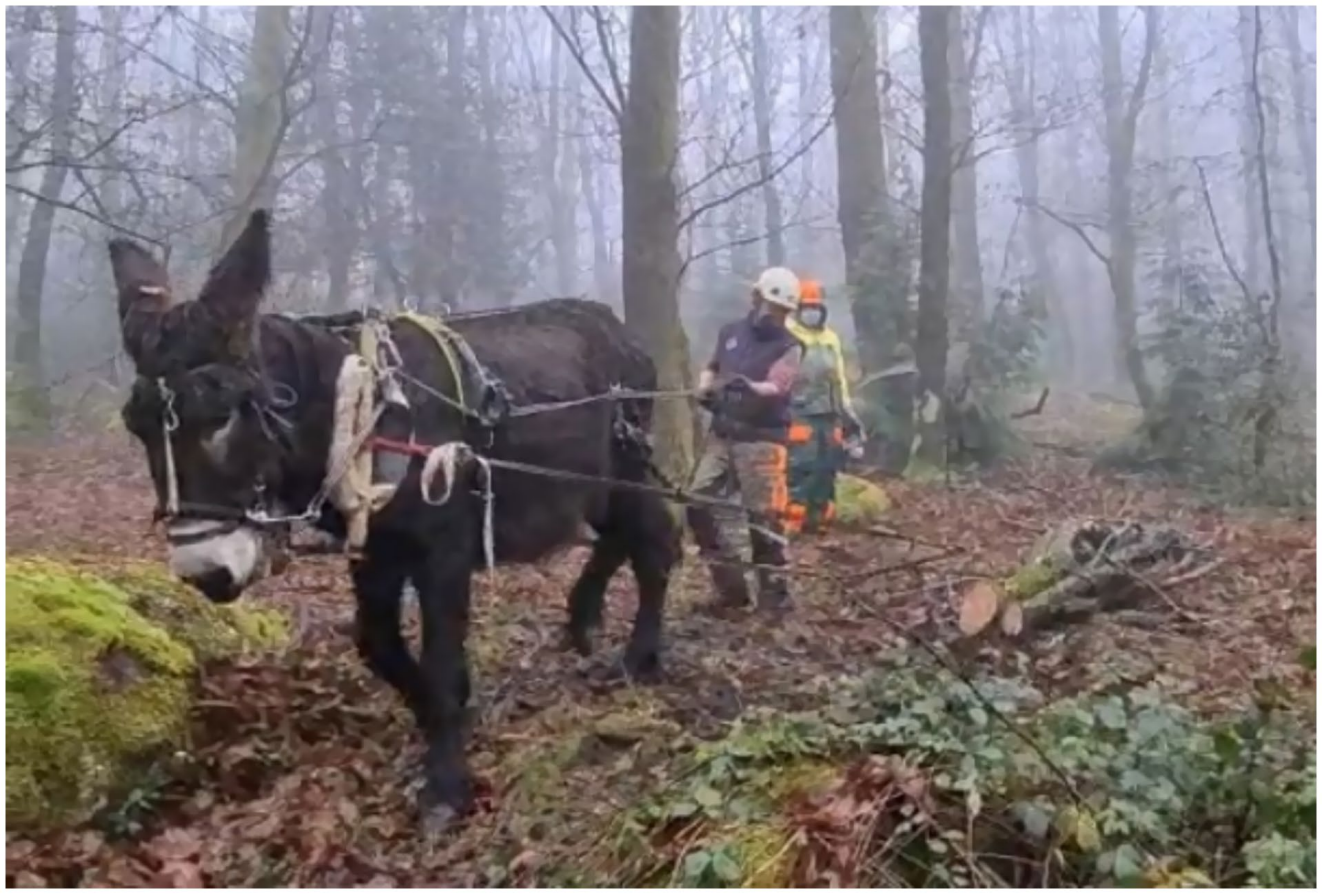
Donkey, Zimbro, labouring in an agro-forestry context. Photo credit: JR.

*A thick mist hangs heavy in the bare trees. The only sounds discernible are the soft snapping of twigs and the churning sounds of chainsaws for which the working equid and his owner show no acknowledgment (Figure* *2**). P1 walks steadily behind his donkey who is harnessed to two short lengths of tree trunk. The procession gently weaves downhill through the woodland, the only marks left behind are from the shallow scraping of the trunks as they transit across the leaf litter. Otherwise, donkey and man leave no trace. A working practice sensitive to the landscape and environment causing minimal soil and plant disturbance and negligible soil compaction. There are no audible sounds coming from the working pair, yet both seem to understand what is required to get the job done.*

*On the uphill return more encouragement is given with a repetitive ‘hup, hup, hup’ to keep momentum to reach where their next load is waiting. P1 says his equids know just how hard they need to pull, depending on where they are, the job role, harness type and terrain. The intensity of vocalisation also highlights the power required, P1 says ‘depending on the intensity of my voice, he will react […] if I put the voice command to walk with more intensity you will see how he goes straight into the collar and he pulls.’*

*Standing patiently waiting for another tree to be attached, the donkey, Zimbro, understands and remembers the work pattern, pull, unload, return, load, pull, unload, return. The job requires steady commitment, so rest is taken by the equid each time a load is hitched before preparing for the next pull. When more power is required two donkeys are hitched together to a small, red metal sled to which a larger tree trunk is attached. P1 moves round to the front of the operation ahead giving verbal encouragement to the working partners to keep going up a steep section with their heavier load. Both donkeys look at ease, taking the strain but looking well within their physical limits. Each partner within the working group knows their part of the job. There seems to be mind-reading or perhaps body-reading as everyone moves around with precision and accuracy, stopping, starting, navigating. There is obvious trust between everyone involved, P1 leaves the donkeys to find their favoured route through the woodland, as P1 says ‘it’s very important that the animals learn that they are actually responsible for the decisions, you have to trust their judgement as the person working with them cannot always see what is ahead.’ The trust in his equids means he listens when they are indicating something, rather than just urging them forwards ‘if your working partners suddenly stop, that’s for sure something in front that you need to go and check’. When asked about the building of trust and the gentle listening involved P1 agreed ‘they wait for your cue, and you wait for theirs’.*

*When giving his working donkeys more autonomy and the freedom to choose to work, P1 says you become attuned to their moods and their language ‘You arrive in the early morning in the stable and after 30 seconds of being with your animal, you say you are not in the mood to work […] it’s not a good day for me to go and work because after 10 minutes we’ll be arguing […] you need to know your animals very well and you need to pay attention to these things’. Although not being economically pressured in the same way as some other work, P1 freely admits, ‘It’s not an emergency, you know, and you decide not to do it […] we could call it the luxury of time […] you are given flexibility’ […] they are not machines, there is always another day’. Making sure all the working conditions are good for the equids, says P1, such as ‘a well fitted harness, good nutrition and health’ means that ‘if that equid does not want to work […] it is not because there’s an illness or painful situation.’*

*Once the equids know the routine for that specific working context they begin to recognise when their working day is ending. If working on the farm, P1 says, ‘they read the soil they are working, they know when the field is finished but when out in the forest they will know when their work is completed as the daylight tells them it’s getting to the end of the day’ (forestry work for P1 is concentrated in autumn and winter when the day length is shorter). When their harness is unhitched, P1 says, his equid’s demeanour and behaviour shifts from ‘full focus to off duty, they down grade their energy’. The equids are slowly walked back to their stable, fed hay to ‘keep them in the stable so any sweat dries before they go out and drink from the spring in the field’. P1 worries that the spring water would be too cold to drink immediately after finishing work while the equids are still hot and sweaty.*

*On rest days all equids are left to always graze out in the fields within their social groups and with access to shelter. P1 feels the relationship with his equids is good and that they enjoy their work because when walking towards the field his working equids will always come to the fence to greet him. P1 says it’s because ‘they know that it’s going to be interaction […] that means that I’m going to take them to another field or that means that I’m going to take them to work’. If one equid is taken to work P1 feels ‘that one goes really happily outside [the field where their companion remains] to work’.*

For centuries equids had been used in Europe to support with the production and transportation of food, wood, and other materials (67, 104–106). As agricultural machinery and modern farming techniques developed, the traditional use of equids dramatically declined in Europe (107, 108). However, there has been a revival in recent years among farmers using equids for agroecological purposes and ‘peasant’ farming techniques (41, 109, 110). These contexts include, vineyards, vegetable gardens, farms/smallholdings and forest management.

The above excerpt is taken from an interview and depicts a particular set of human-equid relations and practices that take place in woodland (being worked as part of a contractual arrangement for its management) and in a smallholding in Paredes de Coura, northwestern Portugal. The smallholding is owned by P1 and his family and managed mainly for self-consumption; any surplus is shared within the local community. It comprises a vineyard and vegetable garden, where all the intermediary work (ploughing and harrowing to prepare the soil) is done with equids, using traditional equipment. In addition to running the smallholding with his family, P1 undertakes agroforestry work for local administrations, providing a service in more sensitive sites (ecologically and/or historically) where heavy machinery is not permitted. For instance, he described a recent commission in a medieval castle where, due to the heritage value of the site, they needed to work sensitively using equids who would tread more lightly on the ground and minimise disturbance. He explained that there is increasing demand for the use of equids in his region, the Iberian Peninsula, with many projects now commissioned by different government bodies, including the European Commission (e.g., the EU’s funding instrument for environment and climate action, EU Life Programme). Where once there were only two or three people undertaking this type of work with equids in his region, he now provides technical training to others, increasing the network of “service providers,” which has positive implications for equid welfare insofar as his equids are not having to travel long distances anymore: “it’s great because I can leave in the morning with the horses, work all day and come back in the afternoon…” Reflecting on the importance of this he says: “People, they do not have yet the sensitivity to understand that the equids are there as living beings and they are my responsibility, and the decisions I take are based on my safety, the team’s safety—but the horses are part of the team you know.” Seeing equids as part of the team acknowledges their labour and, in the case of P1, their individual needs and preferences. And yet it is important to note that P1 is also a qualified veterinarian and equine dental technician; his specialist knowledge, skills and expertise are likely to be contributing factors to the approach he takes with his equids, and the conditions under which they work.

Scholars engaged in animal labour scholarship warn of the commodification of animals, and the extraction of labour value (25, 28, 111–113). While much of the revival of equid use in Europe appears to emerge from moves to resist capitalist forms of production and associated environmental exploitation [e.g., see “back to the land” movement in Europe, (114)] insofar as there is an emphasis on relationships of care, mutuality and the diverse economy (110), it is important to acknowledge the potential neoliberalisation of certain values associated with equid use, particularly instances where equids are constructed as commodity labour or in terms of the value they generate when enrolled within sustainable development agendas. In recent years, working equids in Europe have been described as “clean and renewable power source(s)” (115), as “modern motors” (116), as “green assets” (106), as “resources” (113), with “multiple use values” who can contribute to “financial, ecological and social capital” (114). While each instance of equid use in agroforestry work and subsistence farming in Europe will present a unique set of working conditions and human-equid relations, it is important to recognise that the equids in these contexts are still working and being valued, in part, for the services they provide. Therefore, following others (27, 115) we caution against the construction of animals as equal partners or coworkers in such contexts, instead acknowledging power dynamics at play and attending to the ways in which animals might resist such work.

### Case study 3: ‘affective work’ equine use in equine assisted activities

England, field notes, 12 March 2019, AM.

**Figure 3:**
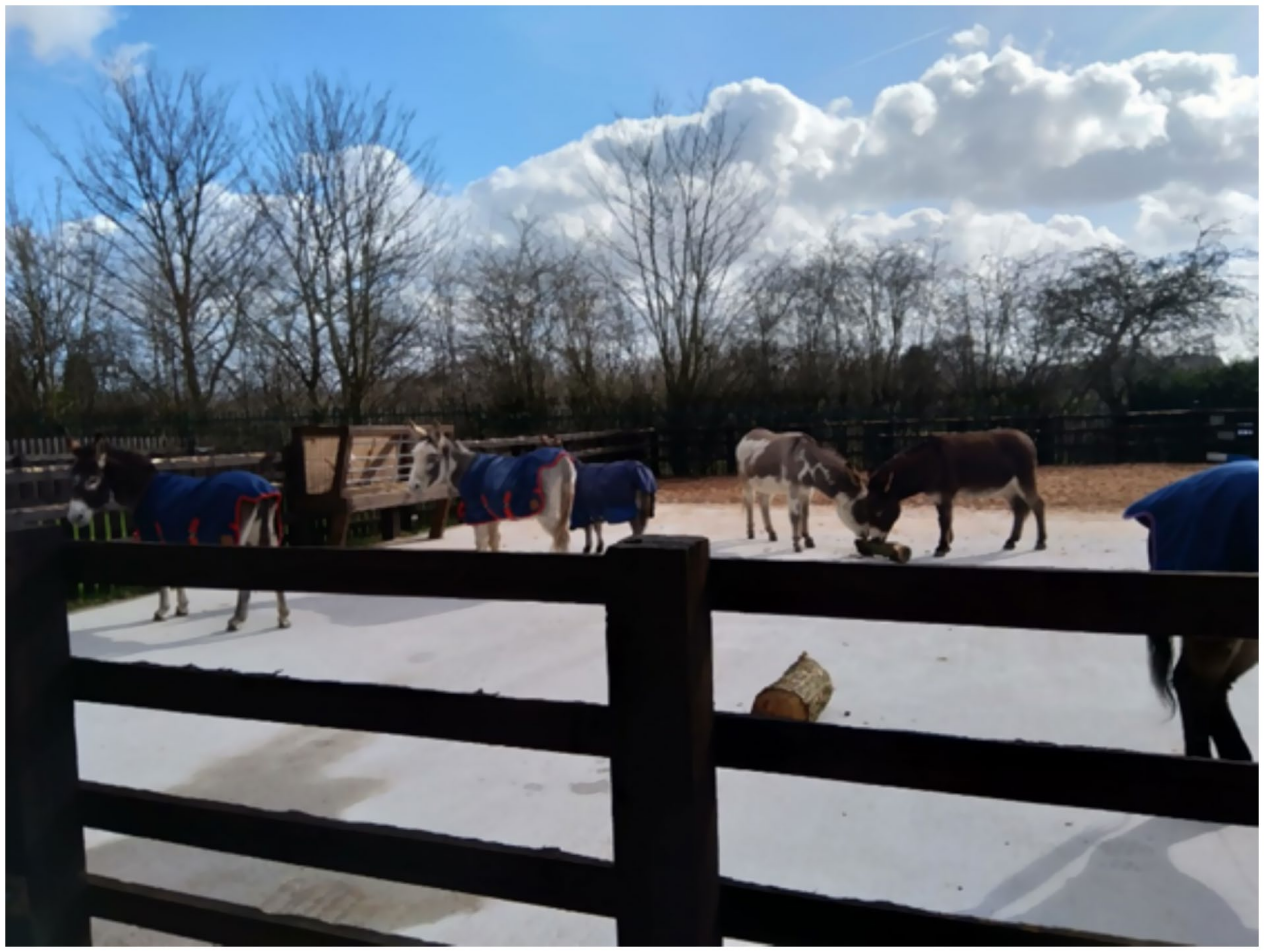
Donkeys having a rest break in between donkey assisted activity sessions. Photo credit: CC.

*We’re outside by the donkey paddocks, surrounded by trees. Residential houses beyond. Several donkeys loiter on the yard, some wrapped up in cosy-looking rugs. It’s sunny but cold, and there’s a strong breeze. Two teenage boys are taking part in the session today. There are normally a few others from their school, but they are the only participants today. They arrive with a support worker from the school who waits inside. The facilitator starts the session with a mindfulness exercise, asking the participants to focus on a spot. After a while, the facilitator invites them to notice any sounds, then any sensations, such as the contact with the ground…I have a go while I’m there. I notice the sun on my face, the sound of dripping water, the birds in the distance. The donkeys are focused on munching their logs, completely absorbed. The boys are silently observing them. At the end of the exercise, the facilitator asks the boys what they noticed. They had clearly been focused on the donkeys the whole time: “They like those logs; they did not even seem to notice us” along with lots of questions: “Why have they got blankets on? Why did that one follow him? Are they friends?” We move inside into the arena (Figure* *3**).*

*The arena is empty other than two donkeys and two donkey assistants (who I’m later told are there to support the donkeys’ experience). The assistants are stationed along the bank of the enclosed area. Inside, it’s completely silent, almost soundproof—even though it’s windy, I cannot hear the wind at all. I sit on the outside the arena, on a viewing platform with a table and chairs. The boys enter the arena, along with the facilitator, and are invited to observe the two donkeys and watch what they are doing. It feels more formal than the exercise outside on the yard, almost like the real work is beginning now. The two donkeys are at the same end of the arena, sussing out their surroundings. The exercise is on the theme of ‘boundaries’ I am later told. The facilitator invites the boys to approach the donkeys and read how they are feeling. As they do so, she asks them to imagine that the donkey has a bubble around him, which is his comfort zone. With the first donkey, one of the boys notices that ‘he seems a bit nervous’. The facilitator asks different questions: “Why do you think that? What do you think it means?. Talk me through what happened? OK, let us rewind a bit. When did he first seem nervous? What did you do when you realised, he was nervous?” They repeat the exercise with the other donkey: slowly walking towards him, assessing his emotional state, reflecting, moving away. The second donkey stands and stares at them, confused, bemused perhaps. The atmosphere, the interactions… it all seems to take a lot of energy and concentration from everyone, including the donkeys. The boys get a bit closer to the donkey. The facilitator asks them what his response is, “What’s he saying?” One boy replies, “he’s alright” and moves to give him a pat.*

*At the end of exercise, they come back together to discuss their experience and reflect on the idea of boundaries and comfort zones: “So how do we know when we have reached that bubble? … What do we do when we reach that bubble?.” The boys commented on what they noticed: “The first donkey seemed really aware of us.” Facilitator: “So do you think different donkeys have different boundaries?” Boys: “Yes” [nodding]. Facilitator: “Why?” The boys respond: “Their personality,” “Like if they are introverted or extroverted,” “How it’s feeling on the day.” Facilitator: “Can a donkey’s boundaries change day to day? What might change their boundaries?.” Boys: “The person they are with. their feelings.” Facilitator: “Could we say the same for people?” Boys: “Yeah. like if you are not feeling good.” Facilitator: “So what donkey are you most like?” First boy “That one,” Second boy: “Dunno, depends on the day.”*

*The session lasted about 20 minutes. Afterwards, the donkeys went for their lunch and the boys returned to school with their key worker. I was tired and I imagined the others felt the same. It was performative in a sense; intentional and orchestrated, yet also spontaneous and intuitive …. I could not help wondering how different they all might be in their normal/natural environments and everyday routines.*

The excerpt above was taken from field notes written during a donkey-facilitated learning (DFL)^7^ session facilitated by The Donkey Sanctuary, an equine welfare charity dedicated to improving the lives of donkeys and mules around the world. The extract demonstrates the kind of mental and emotional efforts that are required for animal assisted activities. The session was intense: full focus was required from everyone, with the participants moderating their behaviour in response to the donkeys, continually analysing and reflecting. Meanwhile, the donkeys were (somewhat awkwardly) entangled in this shared work dynamic, not entirely sure what is expected of them—and yet with some choice to engage or not engage, since they work without headcollars or restraint. Donkey assisted activity (DAA) practitioners interviewed as part of the study described how “the donkeys have to be physically and psychologically well to be able to do this work, because we are asking an awful lot of them” (117) and alluded to the potential risks of emotional burnout. As such, practitioners at The Donkey Sanctuary are trained to monitor the interaction between client and donkey during DAA sessions, noting any signs of anxiety, stress, or tiredness. In addition, the donkeys’ workload is limited and continually rotated throughout the group. Some donkeys might regularly put themselves forward for DAA sessions (e.g., by approaching the clients when they arrive on the yard) because they want human interaction. But in these cases, the practitioners intervene to limit their workload, steering the client to work with a different group of donkeys: “so sometimes, we take over and make a choice for them and say, ‘no, that donkey’s done quite a lot this week’, so it’s sharing the workload so to speak” (Practitioner A). Equally, the practitioners are aware of their donkeys’ preferences such as whether they are content to work with school groups or would prefer a smaller group of adults [detailed in Clancy et al. (117)], so there is flexibility in this particular workplace where “the donkeys are working only where they are comfortable working” (Practitioner A).

After reflecting on working practices and experiences, DAA practitioners were asked about the donkeys’ lives *beyond* the DAA work they do. We were told about their daily routines, which consist of specific mealtimes, grooming, hoof picking, health checks and enrichment activities, such as playing with balls, ropes and old wellies, or the use of herbs and browse strategically placed around the yard, or the use of incense oils (e.g., a drop of peppermint) on their favourite toys. In the summer months, the donkeys can come in and out of the fields as they choose. There is also an ‘enrichment track’ that is sometimes used after DAA sessions with clients: “So if they have had a donkey-facilitated learning session, which can be quite emotional, we’ll try and give them access to that track, and it’s got different enrichment activities [so] that they can just do something a bit different and have that downtime.” (Practitioner A). Practitioners explained after the sessions they are on their own time with no demands or expectations of them: “it’s their choice to go where they want to. There’s no more pressure on them once they have finished the session.” (Practitioner A). Quite often they choose to play with their friends. The practitioners explained that the current group of 21 donkeys are “quite a playful, inquisitive group” now consisting of some younger donkeys (two- and three-year-olds) who can be found “galloping around encouraging some of the others that you do not always see play” (Practitioner A). The dynamics of this group were described as “fluid,” “there’s no hierarchy” (Practitioner B). Some seek human interaction more, while others stay with their friends and their bonded pair.

The case study above offers a particular example of DAA work taking place in an animal welfare-focussed setting. Such a setting affords flexibility in the workplace, both in terms of the duration and type of interactions and in terms of the spaces accessed by the equines (the freedom to come and go). The DAA practitioners themselves are also qualified and experienced professionals, attuned to the needs and behaviours of donkeys. But it is important to note that this structure and set-up may not be representative of all equine-assisted activity/interaction (EAA/I) institutions.^8^ EAA/I still remains unregulated in the UK and elsewhere (118). As Seery and Wells (118) point out, practitioners do not need an equine-based qualification, nor a qualification in animal behaviour to practice or trade.

Despite the burgeoning growth of the animal assisted activity/interaction (AAA/I) industry, the objectives of AAA/I remain largely human-centric (119). Most of the literature on AAA/Is (including literature on equine-assisted activities and interventions) which is often produced by AAA/I practitioners or AAA/I organisations (Kieson (120) unpublished)—focusses on the benefits to humans (117, 121). There is a paucity of research on how animals themselves experience these interventions. Critically reviewing recent literature on AAA/Is (120) found a tendency in studies to emphasise the “mutuality” of the human-animal relationship in AAA/Is, with phrases such as “connection,” “attunement” and “partnership” [even “co-therapist”—(122)] regularly being used. Very few studies offer scientific evidence to support the subjective experience of the animal(s) involved and confirm this so-called mutuality [see also (119)]. Much of this indicates that many practitioners within the AAA/I industry have certain expectations of (and make certain assumptions about) animal participation, choice, and consent in these spaces. In fact, a recent study by Hanrahan and Boulton (123) in which AAI practitioners were interviewed about their motivation for working in the industry found that practitioners felt their animals intuitively “attuned” or “connected” with people. The study also found that most practitioners had limited animal welfare knowledge and that their practice was built almost exclusively on their own experiences. These starting points can have negative consequences for animal welfare and wellbeing, insofar as practitioners may (because of their expectations and beliefs about animals engaged in AAA/Is) overlook instances where animal welfare and/or wellbeing is being compromised. Several potential welfare/wellbeing risks have already been identified in the literature^9^ (121), along with further consideration of equids’ needs beyond the work that they do—namely, freedom, forage and friends (3Fs).

Some efforts are being made. Over the last 5 years, there have been a growing number of papers that have highlighted the need to reevaluate the underlying motivations for involving animals in AAA/I and recognise the impact on animal behaviour, welfare and wellbeing (10, 118, 119, 121, 123–128). Some practitioners are taking seriously the subjective experience of their animals and have measures/practices in place to support a positive experience for the animal. As the above case study demonstrates, questions of animal choice and participation are becoming part of the reflective practice; practitioners are often seeking new/innovative ways of promoting donkey autonomy in sessions: “one of the things we should be promoting is that donkeys should not only be at liberty but should have the ability to remove themselves from situations we put them in” (117). Animal work need not necessarily be negative for the animals involved when questions of choice and participation are foregrounded. Careful consideration of animal needs, natural ethology, and individual preferences can potentially make a huge difference to the lives and lived experience of animals at work, paving the way for the promotion of animal autonomy and wellbeing in the workplace.

## Discussion

The following discussion is designed not to compare one case with another, as the context, conditions and agencies (for human and more-than-human) are completely different. There are varying degrees of cooperation and connection between humans and animals in the contexts of work, as such this paper does not ascribe a positive or negative value to the terms ‘work’ and ‘shared work’. However, it is necessary to explore how different factors do manifest and impact on the experience of equids. As Coulter (1) neatly describes “Animals want to live. They also want to be happy. Animals have minds, bodies, feelings, desires, and relationships that are connected to and affected by, and simultaneously distinct from their labour. This means we must not only consider work but also work-lives and lives.”

Case study one (‘hard work’) demonstrates the importance of socioeconomic context and power dynamics within industrial commodified spaces and how this influences relationships between brick kiln workers and their equids (interspecific), but also between equids themselves (intraspecific). Domesticated animals working in coercive and/or capitalised spaces such as extraction and mining are produced as resource-commodity-labour (103)—as are their human counterparts. In such spaces there exists a shared ‘terrain of difference and imbalanced relations’ where the structural domination of animals may be conjoined with that of humans (129). The case illustrated how brick kiln equids have very little autonomy and often have limited opportunities for close social intraspecific interactions (individuals being tied at distinct intervals along a ground-secured line). Their physiological and behavioural needs are entirely governed by the conditions and political economy of life in a brick kiln (27, 84). The conditions are so severe that although social bonds may be present, the opportunities for expressing them are severely limited during work hours and when tied up after work. If taken to graze by owners, the brief freedom and foraging opportunities afforded may be the donkeys’ primary focus, particularly in the absence of diets providing adequate nutritive and exploratory complexity (14, 130, 131). The transient nature of the brick kiln industry (and with it, the absence of familial/community knowledge transmission) paves the way for a lack of ingrained equid understanding and resulting poor welfare outcomes for those animals (6, 132, 133).

Case study two (‘decent work’) demonstrates how small-scale farming practices and sustainable agroecological work in Europe can create opportunities for attentive human-equid relationships to emerge. In circumstances with less economic and time constraints, it leaves space to support the development of relationships of trust and cooperation (rather than commodification) with the equids but also between people via a supportive network of knowledge sharing. This is why we chose to use the term ‘work’ [See international labour conference in 1999—(2, 38, 134); SDG 8]. As Wadham and Dashper (2) point out, decoupling sustainable development from economic growth (which puts additional pressure on the human and non-human partnership) supports a slower “holistic and all-encompassing” working strategy removing the speed dictated by capitalist demands and replacing it with a re-imagining of “the kind of (shared world) we want to live and work in with our animal neighbours.”

Case study three (‘affective work’) demonstrates that the animals working within the AAA/I industry could benefit hugely from the recognition of their contributions as labour (affective and emotional). In her efforts to conceptualise nonhumans as workers within tourism industries, Dashper (135) describes the emotional labour performed by horses as they interact with their customers. Here they are expected to be calm, friendly, patient, willing—the “trained management of feeling” [(136); cited by Dashper (135)]. According to Dashper (135), these expectations can result in high levels of stress or sanction “such as being told off or ultimately replaced” as Dashper (135) puts it. When human expectations of equids (how they should supposedly perform) are minimised or removed altogether, then animal-centred approaches to AAA/I can flourish. In the case of donkey-facilitated learning at The Donkey Sanctuary, the donkeys were at liberty to walk away at any point—so even if the participants had preconceptions of how the donkeys might interact with them, there was no enforced pressure or expectation. Animal autonomy is possible in these spaces and yet it is hugely contingent upon the expertise and willingness of human practitioners, supported by the absence of human-oriented pressures—whether this be economic, practical (time/space) or the anthropocentric-driven goals of human wellbeing in AAAs/Is. The non-working lives of the donkeys (the opportunities for rest, play, and kinship) were considered a critical part of their overall experience as working animals within this welfare/sanctuary setting. And yet, it is important to continually reflect on what ‘positive welfare’ looks in these complex AAA/I contexts, where subtle emotional pressures can easily arise and shape equid experiences.

By including three different case studies (while not comparing in simplistic ways or elevating one over the other), this paper offers a framework for thinking about the characteristics and qualities of different forms of equid work (‘hard’, ‘decent’, ‘emotional’). The categories were not designed to be prescriptive, i.e., you can have hard work that is also emotionally exhausting for the animal, but we chose them to act as a guide for reflecting on some of the broad characteristics of equid work. For instance, the notion of ‘hard work’ was meant to denote and emphasise the physicality of equid work in tough and often gruelling conditions. Further investigations in this vein might explore the complexity of human-equid relations in the context of work, including the beliefs, attitudes and perceptions of equid owners/handlers towards their working equids, and the multiple identities they hold, e.g., where their equids may be valued both as companions and as sources of livelihood. As this paper demonstrates, human-equid relations and the beliefs, values, and identities that underpin them are influenced largely by context and therefore any categorisation of equid work (as ‘hard’, ‘decent’, ‘emotional’) must attend to these contextual factors.

The case studies presented here demonstrate the importance of relationality—both in terms of actual relations (human-animal) and in terms of context, such as space and environment, socio-cultural factors and political-economic factors—all of which shape the freedoms and opportunities that are afforded to humans and animals in these contexts. In this sense, humans and equids are entangled in shared work dynamics that have significant impacts on their welfare. For this reason, the notion of ‘shared work’ is particularly productive in discussions where contextual factors shape the lives of humans and equids at work. In addition to context, equid-owner dynamics have a critical role to play in shaping equid experiences at work. For instance, Case Study two illustrated that human-equid kinship can flourish when there are fewer economic and/or time pressures and owners/handlers have the capacity to ‘get to know’ the needs and preferences of their equids. Equally, equid knowledge is key. As we stated in the introduction, donkeys can mask signs of stress, fear and fatigue, and so they are vulnerable to overwork/burnout and compromised welfare, unless handlers/owners are particularly attuned to their animals. In the introduction we say that positive equid experiences will partly depend on the knowledge and experience of those they interact with. This does not necessarily have to be technical expertise; lay knowledge can also be the grounding for human-animal kinship (137–139) along with beliefs, attitudes and perceptions of animal sentience (140, 141). But as we know, all of these factors are intertwined with culture and context (27, 84).

Although the focus of this special issue is the 3Fs, we take an expanded view in this paper, using our case studies to highlight the complexities of human-equid kinship; particularly how social, cultural and political-economic contexts intersect with knowledge and/or inhibit opportunities for freedom, forage and friends. We argue that ‘animal flourishing’—borrowing sentiments from ‘positive animal welfare’ agendas (15)—can only truly emerge when we pay close attention to the aforementioned factors. By examining practices of rest, play and kinship in our case studies, we have learnt that human/nonhuman companionship and cooperation can and does exist, but we must be careful not to romanticise interspecies relations or downplay the power dynamics in working equid contexts. In addition to the 3Fs, the article also draws upon the welfare concepts represented within the five domains model (9, 142). We wanted to highlight the importance of knowing individuals, a key component of welfare and wellbeing, and to encourage, where possible, that an equid’s individual preferences are acknowledged, respected and incorporated into their working routines (such as those described within case studies 2 and 3).

For this reason, we made every effort to offer real glimpses into the subjective, embodied, emotional, and relational experiences of nonhuman animals as they work with humans (through our vignettes)—but importantly, we contextualised these writings afterwards. We welcome more work along these lines, building interdisciplinary collaboration and the cross-fertilisation of ideas between welfare science, animal labour scholarship, and those advocating for working equids. We encourage more reflection on “the complex ways that animals choose to participate, or otherwise abstain, from the practices and spaces they are invited into” (117). Resistance to work is a demonstration of animal agency and perhaps, by listening to this, positive experiences for equids can be achieved. However, we appreciate in some contexts, such as those involving ‘hard work’, the opportunities and capacities to do so may be severely limited by the exploitative, coercive labour pressures that are often found in these situations (102).

In conclusion, this paper has demonstrated the value of bringing animal labour scholarship into dialogue with animal welfare debates and principles. It has brought to bear the complexities of equid work, the physical and emotional toil involved, and the inextricable entanglement of equid work with place, space and context. We argue that further work in this area could be hugely beneficial to the working lives of equids around the world, particularly donkeys whose marginalised status means that their needs, welfare and behavioural expressions are often (mis)understood or may be completely overlooked.

## Data Availability

The datasets presented in this article are not publicly available as they contain sensitive material. Requests to access the datasets should be directed to tamlin.watson@thedonkeysanctuary.org.uk.

## Footnotes

^1^The Five Freedoms was the first accepted evidence-based animal welfare framework giving five freedoms first mentioned in the Brambell Report in 1965 and revised subsequently to include: Freedom from hunger and thirst, freedom from discomfort, freedom from pain, injury and disease, freedom to express normal behaviour and freedom from fear and distress.

^2^Five domains model is a welfare assessment framework created in 1994, it integrates physical and behavioural needs of animals and how they relate to their emotional states. The five domains are: nutrition, physical environment, health, behavioural interactions and mental state.

^3^One Health is a unified approach to balance and optimise the health of people, animals and the environment, https://www.who.int/news-room/questions-and-answers/item/one-health.

^4^One Welfare highlights the interconnections between animal welfare, human wellbeing and the environment, and fosters transdisciplinary collaboration to improve welfare globally, https://www.onewelfareworld.org/about.html.

^5^3 F’s are the three main essential overarching needs to support equine physical and mental wellbeing, https://www.worldhorsewelfare.org/advice/the-3fs-friends-forage-and-freedom#:~:text=As%20horse%20owners%2C%20riders%20and,friends%2C%20forage%2C%20and%20freedom.

^6^The notion of ‘animal work’—as a distinct category, worthy of independent exploration – can be traced back to Arluke and Sanders who used the term briefly to mean ‘work *with* animals’.

^7^Donkey-facilitated learning (DFL) sessions are a particular type of donkey-assisted activity (DAA), described on The Donkey Sanctuary’s website. Available at: https://www.thedonkeysanctuary.org.uk/donkey-assisted-activities/our-programmes (Accessed 28/01/2025).

^8^Broadly speaking, equine-assisted activities and interventions (EEAs/Is) involve humans interacting with equines for therapeutic purposes. However, diverse language exists internationally regarding EEAs/Is (143). Latella D, Abrams B. The Role of the Equine in Animal-Assisted Interactions. In: Fine AH, editor. Handbook on Animal-Assisted Therapy. 5 ed.: Academic Press; 2019. p. 133–62.

^9^Potential welfare risks include signal eavesdropping and sensory overload, emotional contagion, fearful responses and physical risk. To overcome these risks Lewis makes recommendations including that only those suitably qualified should take care of AAI animals, there should be supervision of these animal at work. Animals should undergo suitability assessments and training, there should be risk assessments to limit the animal’s AAI interactions. Client’s medical and behavioural history should undergo a full screening before session commencement.
